# Stopover habitat selection drives variation in the gut microbiome composition and pathogen acquisition by migrating shorebirds

**DOI:** 10.1093/femsec/fiae040

**Published:** 2024-03-21

**Authors:** Radosław Włodarczyk, Joanna Drzewińska-Chańko, Maciej Kamiński, Włodzimierz Meissner, Jan Rapczyński, Katarzyna Janik-Superson, Dawid Krawczyk, Dominik Strapagiel, Agnieszka Ożarowska, Katarzyna Stępniewska, Piotr Minias

**Affiliations:** University of Lodz, Faculty of Biology and Environmental Protection, Department of Biodiversity Studies and Bioeducation,, Banacha 1/3, 90-237 Łódź, Poland; University of Lodz, Faculty of Biology and Environmental Protection, Department of Biodiversity Studies and Bioeducation,, Banacha 1/3, 90-237 Łódź, Poland; University of Lodz, Faculty of Biology and Environmental Protection, Department of Biodiversity Studies and Bioeducation,, Banacha 1/3, 90-237 Łódź, Poland; Ornithology Unit, Department of Vertebrate Ecology and Zoology, Faculty of Biology, University of Gdańsk, Wita Stwosza 59, 80-308 Gdańsk, Poland; Forestry Student Scientific Association, Ornithological Section, Warsaw University of Life Sciences, Nowoursynowska 166, 02-787 Warszawa, Poland; University of Lodz, Faculty of Biology and Environmental Protection, Biobank Lab, Department of Oncobiology and Epigenetics, Pomorska 139, 90-235 Łódź, Poland; University of Lodz, Faculty of Biology and Environmental Protection, Department of Invertebrate Zoology and Hydrobiology, Banacha 12/16, 90-237 Łódź, Poland; University of Lodz, Faculty of Biology and Environmental Protection, Biobank Lab, Department of Oncobiology and Epigenetics, Pomorska 139, 90-235 Łódź, Poland; Ornithology Unit, Department of Vertebrate Ecology and Zoology, Faculty of Biology, University of Gdańsk, Wita Stwosza 59, 80-308 Gdańsk, Poland; Ornithology Unit, Department of Vertebrate Ecology and Zoology, Faculty of Biology, University of Gdańsk, Wita Stwosza 59, 80-308 Gdańsk, Poland; University of Lodz, Faculty of Biology and Environmental Protection, Department of Biodiversity Studies and Bioeducation,, Banacha 1/3, 90-237 Łódź, Poland

**Keywords:** avian pathogens, *Charadrii*, gut microbiome, habitat, migration, shorebirds

## Abstract

Long-distance host movements play a major regulatory role in shaping microbial communities of their digestive tract. Here, we studied gut microbiota composition during seasonal migration in five shorebird species (*Charadrii*) that use different migratory (stopover) habitats. Our analyses revealed significant interspecific variation in both composition and diversity of gut microbiome, but the effect of host identity was weak. A strong variation in gut microbiota was observed between coastal and inland (dam reservoir and river valley) stopover habitats within species. Comparisons between host age classes provided support for an increasing alpha diversity of gut microbiota during ontogeny and an age-related remodeling of microbiome composition. There was, however, no correlation between microbiome and diet composition across study species. Finally, we detected high prevalence of avian pathogens, which may cause zoonotic diseases in humans (e.g. *Vibrio cholerae*) and we identified stopover habitat as one of the major axes of variation in the bacterial pathogen exposure risk in shorebirds. Our study not only sheds new light on ecological processes that shape avian gut microbiota, but also has implications for our better understanding of host–pathogen interface and the role of birds in long-distance transmission of pathogens.

## Teaser

Gut microbiota composition and prevalence of bacterial gut pathogens are related to stopover site habitat in shorebirds during autumn migration, suggesting a crucial role of environmental factors in shaping gut microbial community of migratory birds.

## Introduction

The impact of gut microbiota on the physiology and fitness of hosts is a topic of ever-growing interest in studies on animals. Vertebrate gastrointestinal tract is inhabited by a plethora of bacterial taxa (Waite and Taylor 2014, Bodawatta et al. 2022a), which are essential for health of the host organism and, consequently, for its fitness. The gastrointestinal microbiota modulates the functions of an organism in several ways, being crucial in the digestion and assimilation of nutrients (Stevens and Hume 1998), as well as breaking down nondigestible or toxic compounds (Godoy-Vitorino et al. 2008, Zepeda Mendoza et al. 2018). The gut microbiota also affects immunity, constantly stimulating both adaptive and innate components of the immune system and playing an essential role in the development of immunity during the early stages of host ontogeny (Simon et al. 2016, Thaiss et al. 2016, Evans et al. 2017). The gastrointestinal bacterial community also contributes to individual health by restraining the presence of pathogenic microbes via competitive exclusion, effectively preventing them from causing disease (Kamada et al. 2013, McLaren and Callahan 2020). Finally, the microbiota may affect host behaviour (Mayer 2011, Cusick et al. 2021), which has been mostly demonstrated in model organisms under laboratory conditions (Desbonnet et al. 2014, Slevin et al. 2020), but it remains to be studied whether, and to what degree, the microbiota may determine adaptive behavioural and cognitive performance of wild vertebrates (Davidson et al. 2020).

In nonresident birds, seasonal migration between breeding and wintering grounds represents one of the most important challenges in the annual cycle, which shapes their behavior, physiology, and anatomy (Alerstam 1990, Newton 2008, Butler 2016). During premigratory period many species change their diet and use carbohydrates-rich food to accumulate sufficient fat reserves essential to cross geographical barriers (Bairlein and Simons 1995, Ottich and Dierschke 2003, McWilliams et al. 2004). Some internal organs can be reduced, whereas others are expanded to meet physiological requirements of long-distance flights or hyperphagia, which allows rapid accumulation of fat reserves (McWilliams and Karasov 2001, Bauchinger et al. 2005). Other physiological traits, such as oxygen-carrying capacity of blood or intensity of energy production within cells, can also be adaptively adjusted during the migratory period (Weber 2009, Yap et al. 2019). Finally, migratory species can use various habitats or food resources when they stop and refuel at stopover sites scattered along the migration route (Aamidor et al. 2011, Quinn and Hamilton 2012, Lewis et al. 2016). Taking all this into account, it can be expected that migration *per se* and migration-related adaptations should have an impact on the composition of gut microbiota or its rearrangement during the migratory period. For example, diet is considered one of the most important determinants of gut microbiota diversity (Matheen et al. 2022) and fattening during premigratory period or habitat-related shifts in diet composition en route should enhance alterations of gut microbiota in migratory species. Despite these theoretical considerations, our knowledge on the factors that govern microbiome composition in nonmodel long-distance migratory bird species is still scant (e.g. Wu et al. 2018, Trevelline et al. 2023).

Apart from environmental and ecological factors, composition of the gut microbiota should be shaped by basic individual (intrinsic) traits, such as sex or age (Matheen et al. 2022). So far, most information on this intrinsic variation originates from poultry research (van Dongen et al. 2013), but field studies on wild birds supported substantial role of vertical microbe transfer during feeding between hatchings and their parents as the main factor responsible for establishment of gut symbionts (Diez-Méndez et al. 2023). At the same time, little is known about how bacterial communities within the gastrointestinal tract change with age in wild birds after fledging period, but scant evidence suggest that first year/immature birds host less diverse bacterial communities than adult/mature individuals (Kohl et al. 2019, Hernandez et al. 2021).

Since gut microbiota is shaped by a complex network of extrinsic and intrinsic factors, similar processes are likely to drive acquisition and maintenance of pathogenic bacteria in the host digestive tract. In fact, changes of diet or habitats during migration can expose migratory birds to an array of novel pathogens. Empirical studies documenting prevalence of enteropathogenic bacteria within intestinal flora of wild birds are still sparse and mostly focus on the occurrence of specific strains that present a potential health threat to humans or domestic animals (Hubálek 2004, Benskin et al. 2009). Most data on the prevalence of bacterial pathogens in wild birds come from studies on disease outbreaks yielding high mortality rate (e.g. Kirkwood et al. 1995, Pedersen et al. 2003, Niedringhaus et al. 2021). Pathogenic gastrointestinal flora and the processes of disease transmission have been extensively studied in commercially bred poultry (Evans and Sayers 2000, Bang et al. 2003, Kursa et al. 2022), whereas little is known about the source and prevalence of the microbial pathogens in wild-living bird species (Benskin et al. 2009). Hence, the role of wild migratory birds as vectors of disease could be crucial and underestimated. Some of the most extreme long-distance migrants belong to the clade of *Charadrii* shorebirds (Battley et al 2012), which thus have a great potential for pathogen transmission at large, intercontinental geographical scales (Jourdain et al. 2007, Altizer et al. 2011).

Many Palaearctic shorebird species migrate between breeding areas in the High Arctic and wintering grounds in southern Africa (e.g. different *Calidris* species and wood sandpiper *Tringa glareola*), although some others spend winter in the areas that overlap with their breeding grounds (e.g. common sandpiper *Actitis hypoleucos*, common snipe *Gallinago gallinago*, and lapwing *Vanellus vanellus*) (Piersma 2007). Similarly, some shorebirds cover the entire migration distance in only a few long-distance flights, adopting a time-minimizing, but energetically demanding migration strategy (*Calidris* species), while the others use a large number of stopover sites, flying short distances and using the energy-minimization strategy (snipe and lapwings) (van de Kam et al. 2004). Migrating shorebirds prefer different habitat types (e.g. coastal vs. freshwater) and their choice might not only be related to species-specific preferences, but can also be modulated by age, sex, or body condition (van Gils et al. 2000, Piersma 2003, Kober and Bairlein 2009, Allen et al. 2020). As marine/brackish and freshwater habitats can strongly differ in the amount and type of available food supply (Colwell and Landrum 1993, Piersma et al. 1993a), pathogen or predation pressure (Mendes et al. 2005, Rosa et al. 2006), and stability of environmental conditions (Verkuil et al. 1993, Piersma 2012), habitat choice during migration may be crucial not only for shorebird behavior, but also physiology. However, even within similar habitats, shorebirds exploit species-specific food resources (hard to digest bivalves and soft bodied larvae of insects or polychaetes), as different shorebird species show unique adaptations in the morphology of foraging apparatus, e.g. bill shape and length (Barbosa and Moreno 1999). Differences in foraging strategies during migration can also have important fitness consequences, e.g. species exploiting different food resources within similar habitat types can vary in pathogen exposure and infection risk (Clark et al. 2015, Minias et al. 2016).

The main aim of our study was to comprehensively investigate how host species, habitat, and age influence gut microbiomes and bacterial pathogens in migratory shorebird species. To do so, we collected faecal samples from five shorebird species at one coastal site (sandy seashore) and two inland sites (artificial reservoir mudflats and natural riverbed) in Central Europe (Poland). We used bacterial 16S rRNA metabarcoding to obtain data on the composition and diversity of gut microbiome across all our study species and our analyses were targeted at four specific aims. First, we aimed to characterize interspecific differences in gut microbiota, likely reflecting taxonomic variation and species-specific differences in migration and foraging strategies. Second, we characterized intraspecific variation in gut microbiota between different stopover sites and examined whether microbiome diversity and composition were better explained by habitat selection than host taxonomy. Third, we tested for age-related variation in shorebird microbiome and we hypothesized that the gut microbiota of young (immature) birds should be less diverse and significantly different in composition from the microbiome of adult individuals (Somers et al. 2023). Finally, we focused on the presence of pathogenic taxa within the gut microbiota to assess variation in infection risk related to taxonomy, stopover habitat selection, and host age.

## Materials and methods

### Fieldworks

**Figure 1.**
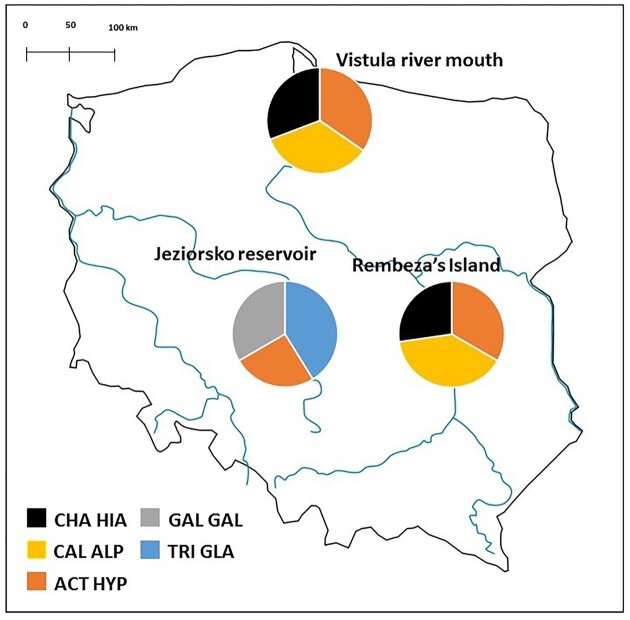
Location of study sites with sampling effort at each location. Species are marked with acronyms: common ringed plover (CHA HIA), dunlin (CAL ALP), common sandpiper (ACT HYP), common snipe (GAL GAL), wood sandpiper (TRI GLA).

The field work was carried out in two consecutive seasons of shorebird autumn migration (June–September 2020 and 2021). Shorebirds were captured at three important stopover sites for migratory waterbirds in Poland: Vistula river mouth (54°21′N, 18°57′E), Jeziorsko reservoir (51°47′N, 18°40′E), and Rembeza’s Island (51°57′N, 21°16′E; Fig. 1). The first study area (Vistula river mouth) was located on the southern Baltic Sea coast, where birds use sandy beaches and small brackish ponds within sandy dunes during foraging and resting. The second site (Jeziorsko) was located at an artificial dam reservoir, where the water level decreases gradually in autumn, exposing extensive areas of mudflats rich in benthic invertebrates. The third site (Rembeza’s Island) was located in the natural Middle Vistula river valley, characterized by the presence of sandy islets, large areas of muddy river banks, and locally shallow riverbed. Thus, each sampling site was associated with a different habitat type (i.e. sea coast, artificial inland mudflats, and natural river valley), representing key stopover habitats used by migrating waders in Central Europe. Birds were captured in walk-in traps designed for small and middle-size shorebirds (Busse and Meissner 2015), aged using plumage characteristics (Demongin 2016), and ringed to avoid repeated sampling of recaptured individuals. Afterwards, each bird was kept in a small cage (48 cm × 31 cm × 33 cm) with a sterilized lining. Birds were released from the cage either after 5 minutes, if no faeces were deposited, or immediately upon defecation. Faecal samples were transferred to sterile Eppendorf tube with 96% ethanol in 2020 or ATL buffer (QIAGEN GmbH, Germany) in 2021. Samples were cooled in portable refrigerators before transportation to the laboratory, where they were kept at −18°C until DNA isolation.

Five different shorebird species were sampled: wood sandpiper (TRI GLA), common snipe (GAL GAL), common sandpiper (ACT HYP), dunlin *Calidris alpina* (CAL ALP), and common ringed plover *Charadrius hiaticula* (CHA HIA). Although we originally aimed to collect samples from each species across different sampling sites, it was not feasible because of different species-specific capture rates at each stopover. We set a threshold of 10 samples collected per species per site and this criterion was satisfied across all three sampling sites for only one species (common sandpiper), across two sites (sea coast and river valley) for another two species (common ringed plover and dunlin), and at only one site (artificial inland mudflats) for the remaining two species (wood sandpiper and common snipe). It was possible to collect good quality samples from the two distinguishable age classes (juvenile vs. adult) only for the dunlin (50% of adults) and common ringed plover (36% of adults). Samples from the remaining species were collected only for juveniles, which are much more abundant during autumn migration than adults (Meltofte 2008).

### DNA extraction, sequencing, and bioinformatics

Bacterial DNA for microbiome metabarcoding was extracted from faecal samples using the DNeasy PowerSoil Pro kit (QIAGEN GmbH) following the manufacturer’s protocol. For the first subset of faecal samples, we used 96% ethanol as a storage buffer, but due to the low efficiency of DNA extraction (only 24% of extracted faecal samples yielded > 10 ng/µl DNA concentration), we decided to store all the remaining samples in the ATL tissue lysis buffer (QIAGEN GmbH), which resulted in a marked improvement of DNA extraction rate using the same extraction kit (99% samples showing > 10 ng/µl DNA concentration). All samples yielding poor DNA extraction success were excluded from downstream processing. However, since storage conditions may affect the composition of bacterial strains detected in avian faecal samples (Zhou et al. 2019), we controlled for sample storage buffer in the analyses of microbiome variation. Sequencing of the variable V3 and V4 regions of the 16S rRNA gene was conducted using primers 515F–806R applied in Earth microbiome project (Klindworth et al. 2013) and standard Illumina protocol, following the Illumina 16S Metagenomic Sequencing Library Preparation Guide. The amplicons were multiplexed with a unique dual-barcode combination used for each sample and sequenced using two 250-bp paired end runs on Illumina MiSeq platform in the BioBank Laboratory, University of Lodz. In total, 180 samples were sequenced, including 159 unique samples, 15 technical replicates (independent amplicons from the same samples replicated within and between sequencing runs, eight and seven samples, respectively), and six negative controls (amplicons from the buffer with no faecal material).

Raw MiSeq sequences were trimmed using Trim Galore v0.6.7. Process of quality filtering, merging, denoising and filtering chimeras was performed with DADA2 algorithm implemented in the QIIME2 v2022.2 package (Bolyen et al. 2019). Sequences were categorized to amplicon sequence variants (ASVs) based on 100% similarity. Bacterial species were recognized based on 97% sequence similarity using Silva 138 SSURef NR99 database (Quast et al. 2013, Robeson et al. 2021). Identification of putative pathogens was conducted using two databases, Silva 138 SSURef NR99 and FAPROTAX script (Louca et al. 2016) and we assigned our bacterial taxa to four ecological groups (human pathogens, intracellular parasites, predatory or exoparasitic, and animal parasites or symbionts). All bacterial species assigned to these groups were verified against classifications provided by Benskin et al. (2009) and other available literature to confirm their pathogenicity in avian hosts. Putative nonbacterial sequences (mitochondrial, chloroplast, and cyanobacteria) were removed from the dataset using QIIME2 filtering plugins. Processing of negative controls revealed little evidence of contamination [on average 292.6 ± 84.8 (SE) reads per sample]. Samples with < 1000 reads were removed from downstream analyses. Processing and analysis of technical replicates indicated excellent replicability of gut microbiome composition. In fact, the relative ASV abundance within each sample (proportion of raw reads of specific ASVs to total number of reads) showed very high and significant intraclass coefficients (ICC), both within and between sequencing runs (ICC > 0.99, all *P* < .001). ICC were calculated using the *irr* R package (Gamer et al. 2019) developed for the R statistical environment (R Development Core Team 2021). In total, 136 samples passed our quality control process and the final sample size ranged from 17 (common snipe) to 42 (common sandpiper) samples [on average 16 ± 1.5 (SE) samples per species per site] [Fig. 1; Table S1 (Supporting Information) in the Electronic Supplementary Material].

Alpha diversity (ASV richness and Shannon diversity index H’) and beta diversity (pairwise Jaccard distances) indices were calculated using the BIOM table in QIIME2 to evaluate richness and diversity of bacterial species. Microbiome composition was characterized at different levels of taxonomic resolution (phylum, class, and genus) using the mean number of reads assigned to each taxon relative to the total number of reads. We used rarefaction (as implemented into QIIME2) to normalize data (diversity measures) for variation in sequencing depth (i.e. number of sequences) (Weiss et al. 2017).

General diet composition of our study shorebird species was characterized using data summarized by Cramp et al. (1998). For each species, we compiled information on the presence or absence of main prey types in the diet and this characterization was conducted on the higher (class) and lower (family) taxonomic level. We used Jaccard distance to quantify pairwise diet differentiation between species.

### Statistical analysis

To compare the gut microbiome alpha diversity between species, sites, and age classes, we entered ASV richness and Shannon index H’ as the response variables in separate generalized linear models (GLMs). First, we ran GLMs testing for interspecific differences in alpha diversity and species identity was entered as a fixed factor in these models. In the second step, species-specific models were developed for three species that were sampled across different stopover sites (common sandpiper, dunlin, and common ringed plover). In all these models, site was entered as a fixed factor, whereas age was entered as another fixed factor only for dunlin and common ringed plover (all sampled common sandpipers were juveniles). In all GLMs storage buffer type was included as a fixed factor to control for any possible variation in microbiome diversity and composition attributed to different sample storage protocols. ASV richness was analysed using Poisson distribution models and a logit link function, whereas H’ was analysed using Gaussian distribution models. To compare the gut microbiome composition between species, sites, and age classes, we entered beta diversity indices (Jaccard distance matrices based on the presence/absence of ASVs) into permutational multivariate analysis of variance (PERMANOVA). Three separate models were run with species, site, and age (where possible) as explanatory variables. All PERMANOVA analyses were conducted using the QIIME2 package. Statistical significance was inferred based on the pseudo-F statistic and the resulting *P* values were corrected for multiple testing (*q*-values) using Benjamini and Hochberg method (Benjamini and Hochberg 1985). Differential abundance analysis was conducted using analysis of composition of microbiomes in QIIME2 to identify significantly and differentially abundant ASVs in each sampling location and age group (Mandal et al. 2015). To test for differences in the prevalence of putative pathogenic gut bacteria between species, stopover sites, and age classes, we ran GLMs using binomial distribution and a logit link function. An occurrence of each putative pathogen was entered as a binary response variable (presence/absence) in a separate model. We performed GLM analyses only for pathogens with > 20% total prevalence across all samples. The results of full models are presented for each analysis. The models were fitted using restricted maximum likelihood and run with *glm* function in R statistical environment.

In order to characterize the role of major pathogens in the gut microbial communities we analysed microbial co-occurrence networks (Coyte et al. 2015). Specifically, we estimated the number of interconnections between each major pathogen and other bacterial species that showed significant co-occurrence within samples (Spearman’s correlation coefficient > 0.3). The analysis was performed using networkX and pandas libraries in Python v3.9.2. To compare and visualize composition of microbiome similarity across species and stopover sites, we used nonmetric multidimensional scaling (NMDS) based on Jaccard distances matrix (Hancock 2004). The analysis was performed only for statistically significant relationships, as previously inferred with GLMs models. For the purpose of analysis, we used metaNMDS function from the *vegan* R package. Associations between pairwise differentiation (Jaccard distances) of microbiome and diet composition were analysed with Pearson product-moment correlation coefficient. All values are reported as means ± SE.

## Results

### Composition of gut bacterial communities

**Figure 2.**
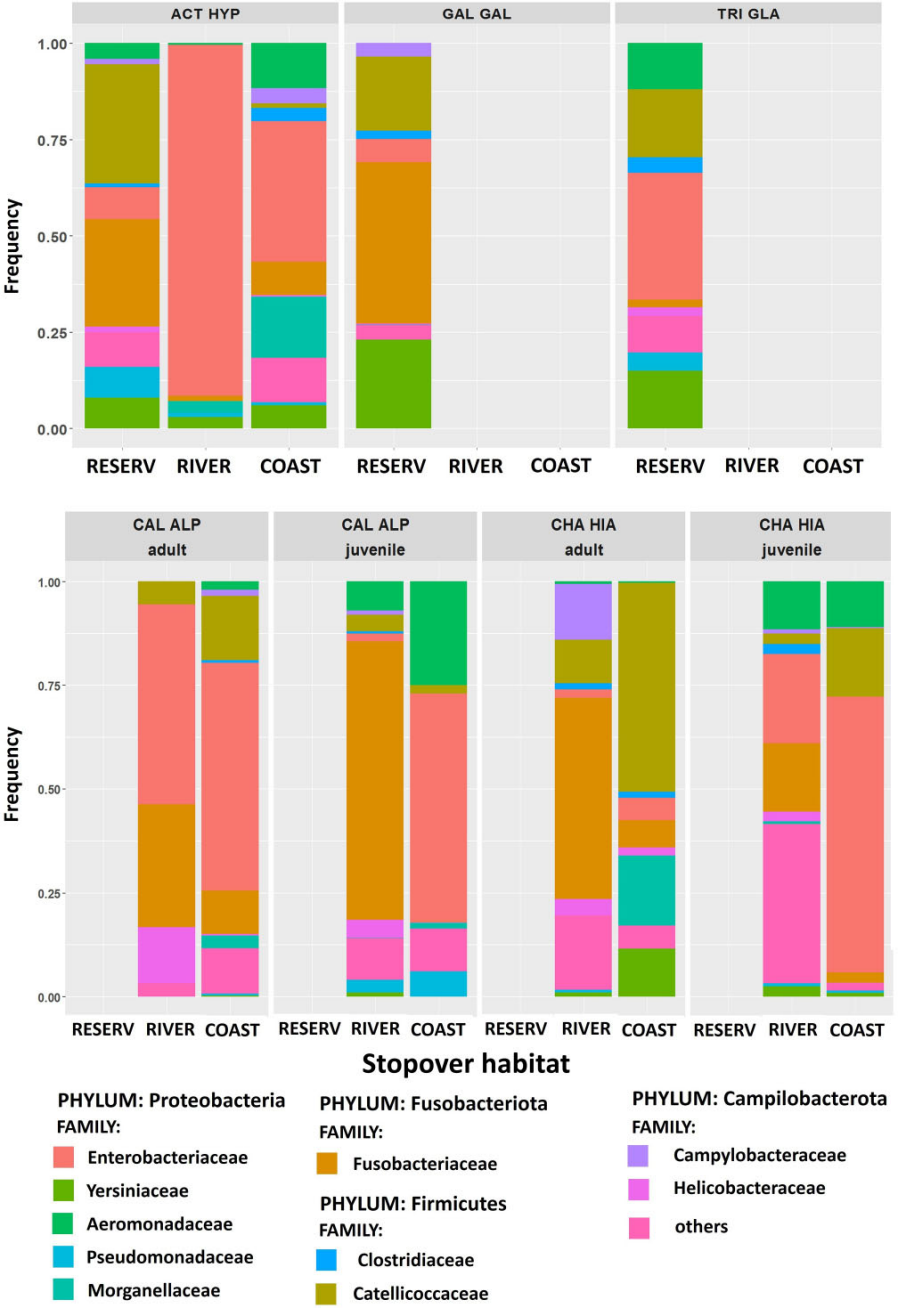
Family-level bacterial composition (relative number of reads) of gut microbiome for five shorebird species migrating through three different stopover habitats, including inland reservoir (RESERV), river valley (RIVER), and sea coast (COAST). Species are marked with acronyms: common ringed plover (CHA HIA), dunlin (CAL ALP), common sandpiper (ACT HYP), common snipe (GAL GAL), and wood sandpiper (TRI GLA).

We detected a total of 39 bacterial phyla in the faecal samples across all five shorebird species (mean 9.51 ± 0.49, range: 2–31), but nine phyla were identified as singletons (detected in one individual). The gut bacterial communities were dominated by the following phyla: *Firmicutes* (prevalence 100%, mean relative abundance 19 ± 2%), *Proteobacteria* (prevalence 100%, mean relative abundance 56 ± 3%), and *Fusobacteriota* (prevalence 82%, mean relative abundance 18 ± 2%; Fig. 2). *Bacilli* and *Clostridia* were the most prevalent classes in the phylum *Firmicutes* (prevalence 98.5% and 95%, respectively). *Proteobacteria* were mainly represented by classes *Gammaproteobacteria* (100%) and *Alphaproteobacteria* (80%), whereas *Fusobacteriota* were mainly represented by class *Fusobacteria* (82%). At the genus level, the most prevalent were *Aeromonas* (76%) and *Enterobacter* (67%) (phylum *Proteobacteria*); *Cetobacerium* (74%) and *Fusobacterium* (65%) (phylum *Fusobacteriota*); and *Catellicoccus* (85%) and *Tyzzerella* (64%) (phylum *Firmicutes*). In total, 8681 ASVs were identified across all samples (mean 128.66 ± 14.76).

### Diversity of the gut microbiome

#### Alpha diversity

There were significant interspecific differences in the gut microbiome alpha diversity, as quantified with ASV richness and H’ (Table S2, Supporting Information). The highest ASV richness (185.0 ± 59.3) was found in the common ringed plover and it was significantly higher when compared to all the other species (range 68.9–164.6, all *P* < .001; Figure S1A, Supporting Information). An analysis of H’ revealed a slightly different pattern, showing similar alpha diversity between common ringed plover, dunlin, and common sandpiper (Table S2 and Figure S1B, Supporting Information), while significantly higher H’ was found in dunlin compared to wood sandpiper and common snipe. The lowest ASV richness was found in the common sandpiper (68.9 ± 8.9), whereas wood sandpiper had lowest H’ (2.45 ± 0.18).

At the intraspecific level, we found significant between-site differences in ASV richness in two shorebird species: common sandpiper and common ringed plover (Table 1). Common sandpipers captured at the inland artificial reservoir had lower microbiome ASV richness compared to birds from coastal areas, but higher than individuals captured in natural river valley (Table 1). In contrast, common ringed plovers from the coastal site had significantly lower ASV richness compared to plovers from natural river valley (Table 1). An analysis of the Shannon index did not support significant between-site variation in the common sandpiper, although it was nearly significant in the common ringed plover, revealing the pattern of variation consistent with ASV richness (Table S3, Supporting Information). No significant between-site differences in microbiome alpha diversity (ASV richness and H’) were found in the dunlin (Table 1; Table S3, Supporting Information).

**Table 1.** Habitat and age variation in ASV richness of the gut microbiome in three shorebird species. Sample storage buffer was included as a fixed factor. Significant predictors are marked in bold.

Species	Predictor	β ± SE	*t*	*P*
Common sandpiper	ASV richness			
	**Intercept**	**4.181 ± 0.035**	**120.53**	**< .001**
	**Site (sea coast vs. reservoir)**	**0.118 ± 0.042**	**2.83**	**.004**
	**Site (river valley vs. reservoir)**	**−0.353 ± 0.056**	**−6.26**	**< .001**
	**Buffer (EtOH vs. ATL)**	**0.568 ± 0.051**	**11.18**	**< .001**
Dunlin	ASV richness			
	**Intercept**	**4.287 ± 0.042**	**101.10**	**< .001**
	Site (river valley vs. sea coast)	0.065 ± 0.048	1.36	.172
	**Age (adult vs. juvenile)**	**0.348 ± 0.041**	**8.58**	**< .001**
	**Buffer (EtOH vs. ATL)**	**1.059 ± 0.039**	**27.32**	**< .001**
Common ringed plover	ASV richness			
	**Intercept**	**4.024 ± 0.033**	**121.18**	**< .001**
	**Site (river valley vs. sea coast)**	**0.406 ± 0.084**	**4.84**	**< .001**
	**Age (adult vs. juvenile)**	**0.599 ± 0.030**	**19.83**	**< .001**
	**Buffer (EtOH vs. ATL)**	**1.420 ± 0.081**	**17.53**	**< .001**

We also found significant age-related variation in the microbiome ASV richness of both common ringed plover and dunlin (i.e. the only two species with both age classes sampled). The patterns of variation were consistent across the species, as adult birds showed significantly higher ASV richness in comparison to juveniles (Table 1). However, no significant age-related variation in the H’ was found in either plovers or dunlins (Table S3, Supporting Information).

#### Beta diversity

**Figure 3.**
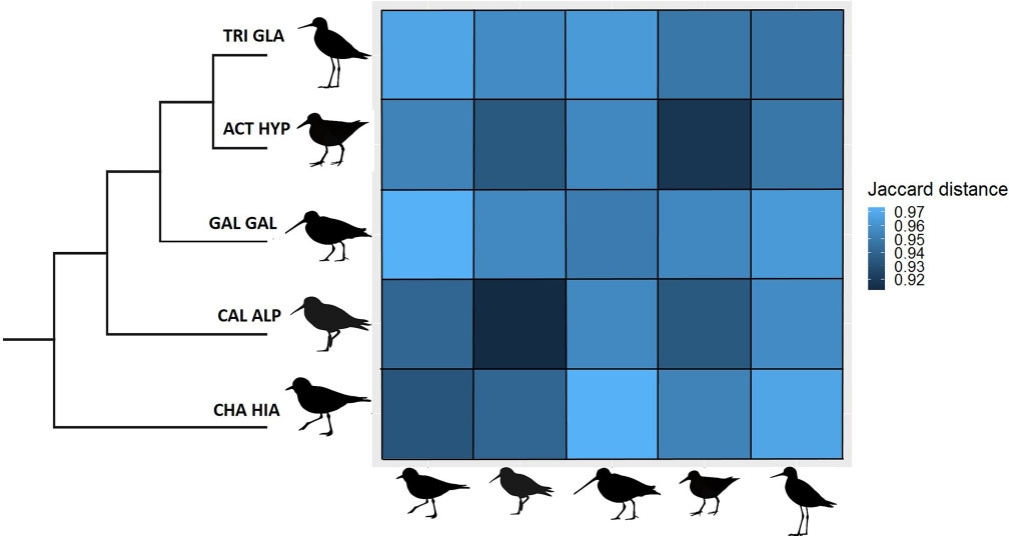
Heatmap showing gut microbiome beta diversity values (mean pairwise Jaccard distances) in five shorebird species migrating through Poland. Phylogenetic relationships between taxa were retrieved from Birdtree webserver (Jetz et al. 2012). Species are marked with acronyms: common ringed plover (CHA HIA), dunlin (CAL ALP), common sandpiper (ACT HYP), common snipe (GAL GAL), and wood sandpiper (TRI GLA).

**Figure 4.**
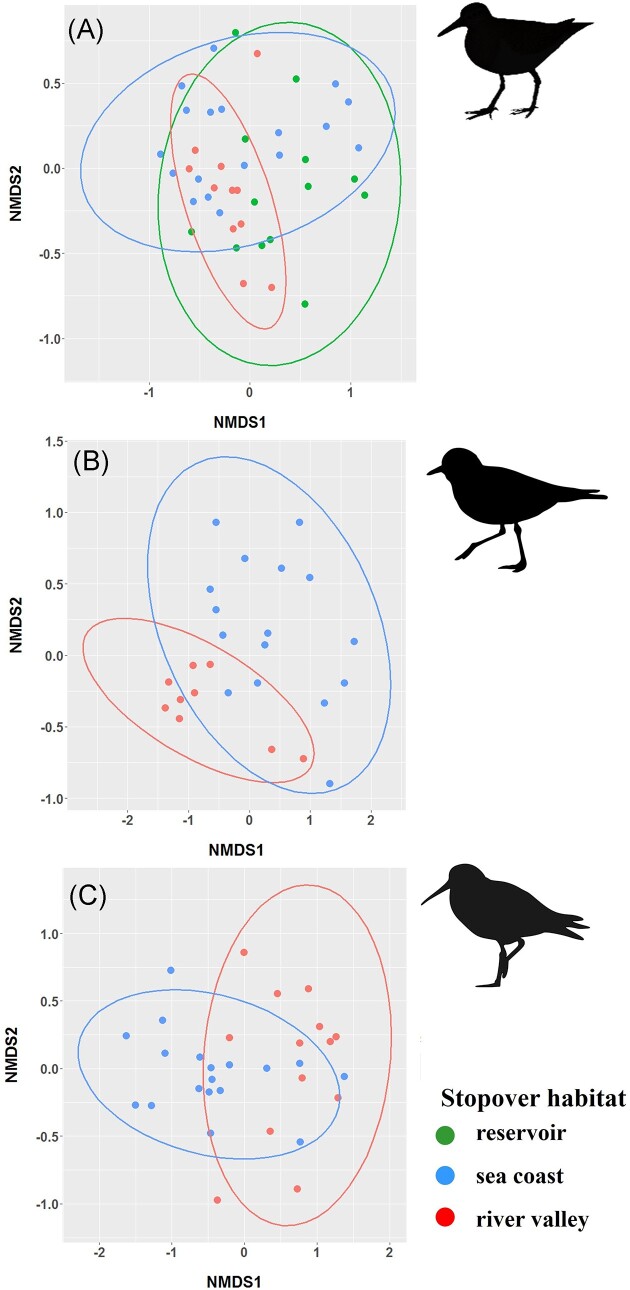
NMDS plot based on Jaccard distances showing the clustering of gut microbiota communities by stopover site for the common sandpiper (A), common ringed plover (B), and dunlin (C).

We found significant taxonomic variation in microbiome beta diversity (PERMANOVA: pseudo-F = 2.29, *P* = .001) and all pairwise comparisons between species were highly significant (*q* < 0.001; Fig. 3). The highest pairwise Jaccard distances were found in the common ringed plover versus common snipe (0.956 ± 0.001) and wood sandpiper (0.957 ± 0.001), while the lowest Jaccard distances were found in the dunlin versus common sandpiper (0.935 ± 0.001) and ringed plover (0.941 ± 0.001). Despite these significant differences, no clear clustering by species was revealed by NMDS approach (Figure S2, Supporting Information). We also found a significant effect of stopover habitat on beta diversity in all shorebird species that were sampled across multiple locations (PERMANOVA: all *P* < .001). When we looked at differences between habitats in the dunlin and common ringed plover, Jaccard distances between river valley and sea coast were significantly higher than within-habitat distances (all *q* < 0.01). In the common sandpiper, the highest Jaccard distances were found between sea coast and inland reservoir (0.949 ± 0.001), whereas the lowest Jaccard distances were found between sea coast and river valley (0.894 ± 0.004). All pairwise between-habitat distances were significantly higher than respective within-habitat distances in this species (all *q* < 0.05). Overall, mean between-habitat Jaccard distances were higher in the common ringed plover (0.946 ± 0.001) compared to dunlin (0.935 ± 0.001) and common sandpiper (0.923 ± 0.002). Consistently, differential abundance analysis revealed the presence of habitat-specific bacteria only in the common ringed plover, including three taxa from *Bacilli* (*Bacillacae*) and *Gammaproteobacteria* (*Steroidobacteraceae* and *Sutterellaceae*) significantly more abundant in samples from river valley than sea coast. NMDS approach showed relatively good clustering of the gut microbiome communities by stopover site within all three species (Fig. 4). Finally, beta diversity was associated with age in the dunlin (PERMANOVA: pseudo-F = 1.61, *P* = .006), although differential abundance analysis failed to identify any specific bacterial taxa significantly contributing to these variation. No age-related variation in beta diversity was detected in the common ringed plover (PERMANOVA: pseudo-F = 1.04, *P* = .28).

### Diet and microbiome composition

The strongest differentiation of diet composition (prey types characterized at the family level) was observed between the common ringed plover and three other species, i.e. common snipe, wood sandpiper, and common sandpiper (Jaccard distance range = 0.90–0.92), while the weakest differentiation was found between common sandpiper and both common snipe and wood sandpiper (Jaccard distance = 0.70). However, when diet was characterized at the higher (class) taxonomic level, common ringed plover showed the lowest differentiation from dunlin (Jaccard distance = 0.20). Despite clear variation in diet composition, there was no significant correlation between the level of interspecific differentiation in diet and microbiome composition, although a positive correlation for diet characterized at the higher (class) taxonomic level approached significance (family level: *r* = 0.50, *P* = .13; Figure S3A, Supporting Information; class level: *r* = 0.60, *P* = .067; Figure S3B, Supporting Information).

### Pathogenic bacteria within the gut microbiome

**Figure 5.**
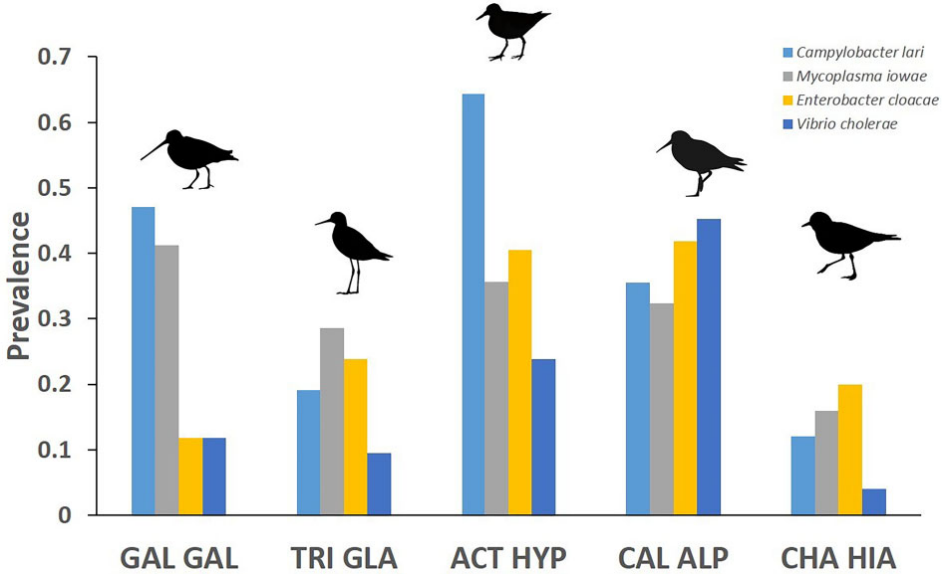
Prevalence of four putative avian pathogenic bacteria in the gut microbiome across five shorebird species migrating through Poland. Species are marked with acronyms: common ringed plover (CHA HIA), dunlin (CAL ALP), common sandpiper (ACT HYP), common snipe (GAL GAL), and wood sandpiper (TRI GLA).

Based on classification from Benskin et al. (2009), we identified 26 species of putative avian pathogenic bacteria in the gut microbiome across all five shorebird species (Table S4, Supporting Information). Additional 33 putative pathogens were identified based on FAPROTAX database (Table S5, Supporting Information), although two of them (*Citrobacter freundii* and *Escherichia coli*) were excluded from downstream analysis despite high prevalences (46.3% and 56.6%, respectively), as showing only opportunistic or facultative pathogenic activity. Nearly half of all identified pathogens (*n* = 26) were present only in a single sample, but eight bacterial species were found in all study shorebird taxa (Tables S4 and S5, Supporting Information). Three pathogenic bacteria (*Campylobacter lari, Mycoplasma iowae*, and *Enterobacter cloacae*) showed relatively high prevalence (> 30%) in the gut microbiome across all hosts (Table S6, Supporting Information; Fig. 5). There were significant interspecific differences in the prevalence of three pathogenic bacteria, i.e. *C. lari, E. cloacae*, and *Vibrio cholerae* (Table S6, Supporting Information). Prevalence of *C. lari* was highest in the common sandpiper and common snipe (64.3% and 47.1%, respectively), showing significant differences from the remaining shorebird species (Table S6, Supporting Information). In contrast, prevalence of *E. cloacae* and *V. cholerae* was significantly higher in the dunlin and common sandpiper, compared to the other three species (Table S6, Supporting Information). *Mycoplasma iowae* was identified to be the most embedded into the co-occurrence network, showing numerous (*n* = 92) positive interconnections with nonpathogenic gastrointestinal bacteria (Figure S4, Supporting Information). *Enterobacter cloacae* showed much fewer interconnections with other members of bacterial community (some shared with *M. iowae*), representing either positive (*n* = 4) or negative (*n* = 4) associations. *Vibrio cholerae* and *C. lari* also showed few interconnections (all positive), but formed separate clusters from *M. iowae* and *E. cloacae* (Figure S4, Supporting Information).

We found considerable between-site variation in the prevalence of pathogenic bacteria, but these patterns were host-specific. The prevalence of *E. cloacae* differed significantly between stopover habitats in only one species, the common sandpiper, as birds from natural river valley were more often infected than birds from artificial reservoir (Table S7, Supporting Information). The prevalence of *M. iowae* differed significantly between stopover habitats in the common sandpiper and dunlin (Table S8, Supporting Information), being higher in common sandpipers from the inland artificial reservoir (61.5%) compared to sea coast (16.7%) and in dunlins from natural river valley (69.2%) compared to the coastal stopover site (5.6%). There were also significant between-site differences in the prevalence of *V. cholerae* in the common sandpiper, but the pattern was opposite to *M. iowae*, as more individuals from the sea coast were infected (50.0%) compared to artificial reservoir (7.7%) (Table S9, Supporting Information). The prevalence of *C. lari* did not show any significant differences between sites (Table S10, Supporting Information). We found significant differences in infection rate between age classes for only one putative bacterial pathogen in only one shorebird species, as the prevalence of *V. cholerae* was significantly higher in adult than juvenile dunlins (81.2% vs. 6.6%, respectively; Table S9, Supporting Information).

## Discussion

Our study not only revealed significant interspecific differences in the composition and diversity of the gut microbiota among five shorebird species during migration, but also provided evidence for intraspecific variation in the gut microbiome between stopover sites and age classes. We also detected the presence of nearly 60 putative bacterial pathogens in shorebird faecal samples, also showing site- and age-related differences in prevalence. Our study reinforces the view that an application of metabarcoding approaches to characterize microbiomes of nonmodel vertebrate species may still provide novel insights into the complex interface between bacterial symbionts/pathogens and their hosts.

Shorebirds are a diverse group showing a high level of variation in terms of ecology, feeding behavior, and habitat preferences (del Hoyo et al. 1996). Their interspecific variation in foraging techniques drives strong differences in diet and eventually may promote interspecific variation in the composition of gut microbiota, even at the same geographical locations. The species-specific composition of gut microbial community in similar environmental conditions was previously found in two shorebird species, the red knot *Calidris canutus* and ruddy turnstone *Arenaria interpres* migrating through Delaware Bay, United States (Grond et al. 2014). In fact, only 5 of 46 bacterial genera were shared between these two species, however, the results were based on material collected only from three individuals (Grond et al. 2014). Similar effect of host taxonomy on the composition of gut microbiota was reported in a wide range of other avian lineages, including ducks, owls, penguins, and passerines (Dewar et al. 2013, Bodawatta et al. 2018, Hird et al. 2018, Maraci et al. 2021, Bartlow et al. 2022). In many cases, differences in the gut microbiota were still apparent despite sympatric occurrence and similar habitat types used by different species (Yang et al. 2016, Cho and Lee 2020, Lu et al. 2022 but see Grond et al. 2019), which is consistent with our results. It has also been suggested that diet and feeding preferences are among the most important drivers of interspecific variation in the composition of gut microbiota, not only in shorebirds, but also in other avian lineages (Grond et al. 2018, Matheen et al. 2022, Sun et al. 2022). This hypothesis has been supported by the observations of diet-related shifts in the gut microbiome composition, as reported for both nonpasserine (e.g. Siberian crane *Grus leucogeranus*, common crane *Grus grus*, and great bustard *Otis tarda dybowskii*; Li et al. 2021, Wang et al. 2023) and passerine birds (e.g. great tit *Parus major* and house sparrow *Passer domesticus*; Davidson et al. 2020, Teyssier et al. 2020). In our study, we did not found a clear statistical support for associations between differentiation in diet and microbiome composition (although some positive trends were detected), but our quantification of diet composition was rather rough and based on published species-specific information. We acknowledge that direct empirical data on individual variation in diet composition could enhance the power to detect microbiome–diet associations (Bodawatta et al. 2022b). The strongest differentiation of the microbiome composition (as expressed by pairwise Jaccard distance) was found between the common snipe and common ringed plover, birds that use completely different feeding techniques (van de Kam 2004 et al. 2004) and exploit different food resources (as shown here with large Jaccard distances observed for main prey types). The common snipe has the longest bill and feeds mainly on large invertebrates (such as earthworms) by probing in mud or soil, whereas the common ringed plover has short pointed bill and feeds on small insects, catching them actively from the surface of water and land (Cramp et al. 1998, Kozik et al. 2022). Despite these differences, we found that many gut microbial taxa were still shared between the hosts and clustering methods did not allow for any clear separation of microbiota according to host taxonomy, suggesting that these effects are relatively weak.

Apart from the taxonomic effects, we observed a substantial environmental component (i.e. stopover site habitat-related variation) in the composition of gut microbiota in all three shorebird species sampled at different locations. Wetland habitats exploited by shorebirds represent a broad gradient of varying environmental conditions, either in terms of sediment quantity, percentage of organic matter, or water quality. Consequently, different wetland ecosystems are inhabited by different invertebrate communities, which requires adjustments in shorebird diet. In general, dam reservoirs located on large lowland rivers in Central Europe are rich in organic sediments and their benthic fauna is often dominated by *Diptera* larvae (mainly *Chirominidae*) and oligochaetes (Grzybkowska and Dukowska 2001, Poznańska et al. 2009). Natural river valleys are more variable in terms of oxygen conditions and sediments, so their benthic fauna is often more diverse compared to anthropogenic reservoirs or regulated rivers (Horsák et al. 2009, Jones 2013). Finally, coastal areas dominated by brackish water and sandy sediments are expected to show high divergent invertebrate faunas from inland wetland ecosystems, being dominated by molluscs, polychaetes, aquatic arthropods, and crustaceans (Kotwicki 1997, Hansen et al. 2008, Henseler et al. 2019). Differences in available food resources may not only impact diet composition of shorebirds that use different wetland ecosystems along their migratory route, but birds at different stopover sites may also ingest different food-associated microorganisms, which could colonize their gastrointestinal tract (Grond et al. 2018). So far, tight relationships between diet, food niche, and microbiome composition were revealed in a comparative study of 21 tropical bird species (Bodawatta et al. 2022b). If diet shapes the gut microbial community, bird species that use different habitats and variable food resources are expected to show higher plasticity in microbiome composition than species adapted to a single habitat type and having narrow foraging niches. We can assume that shorebirds using different stopover sites during migration should respond quickly to local conditions and adjust composition and diversity of their gut microbiota. Extraordinary capacity of birds, shorebirds in particular, for a quick adaptation to local conditions during migration is well acknowledged (Alerstam 1990, Berthold et al. 2003). Migrating shorebirds have been reported to rapidly remodel many physiological processes, such as the rate of nutrient circulation in blood (Jenni-Eiermann and Jenni 2003, Klaassen et al. 2012, Araújo et al. 2022) or fat accumulation rate (Maillet and Weber 2006, Araújo et al. 2019), but they also show rapid morphological adjustments, e.g. temporal shifts in the size of internal organs (Piersma et al. 1993b, Battley et al. 2000). It was also shown that rapid body mass gain during migration in shorebirds was associated with changes in their gut microbiome composition (Grond et al. 2023). An active migration was associated with changes in the gut microbiota in *Calidris* shorebirds. For example, migrating red-necked stints *Calidris ruficollis* had higher prevalence of *Corynebacterium* bacteria than resident conspecifics and similar adjustments were also reported for migrating curlew sandpiper *Calidris ferruginea* (Risely et al. 2018). So far, the majority of studies on habitat-related variation in the gut bacterial community were based on the samples collected at a single location or during the breeding season (e.g. Lewis et al. 2016, Góngora et al. 2021, Drobniak et al. 2022), although some exceptions occur. Between-habitat comparison of the gut microbial community in nine species of Darwin’s finches showed that lowland versus highland habitat variation was a primary determinant the microbiome composition (Loo et al. 2019). Interspecific comparisons also indicated that local environmental conditions may be considered a crucial driver of the gut microbiota composition in birds and their importance may even exceed the effects of phylogenetic relationships (Hird et al. 2014, Grond et al. 2019, Skeen et al. 2023). In agreement with this hypothesis, we observed a relatively good clustering of individual microbiome composition by the stopover site habitat (within species), and this clustering was more apparent that the clustering by species. Similar variation was observed in the microbiome alpha diversity measures, as the common ringed plover and common sandpiper showed different ASV richness between all sampling locations. Thus, habitat type and diet may not only affect taxonomic composition of gut microbiota, but also its overall level of diversity.

So far, age-related differences in the gut microbiota were detected in various bird species, but the majority of studies were based on comparisons between nestlings and adult birds (van Dongen et al. 2013, Barbosa et al. 2016, Bartlow et al. 2022). In general, bacterial community in chicks is often less abundant and more temporarily unstable than in adult individuals (Sun et al. 2022). Highly diverse communities of microbial commensals can improve health status of their host by protecting it from the colonization of intestinal tract by pathogens (Grond et al. 2018). Adult birds with a well-developed immune system and a better resistance to disease than immature individuals are expected to have a diverse community of bacterial commensals. Higher diversity of cloacal microbiota during the breeding season was found in adult (after second year) female tree swallows *Tachycineta bicolor* compared to young individuals (second-year) (Hernandez et al. 2021). The analysis of the gut microbiome composition in mute swans *Cygnus olor* from different age classes also provided evidence for lower diversity of bacterial community in the first year birds compared to older individuals, but these differences were only weakly pronounced (Hill et al. 2023). In our study, first-year dunlins and common ringed plovers had lower gut bacterial diversity in comparison with adult birds, although these differences were apparent only at the level of ASV richness (but not H’). Interestingly, not only alpha diversity, but also the composition of gut microbiota showed age-related differences in the dunlin. It suggests that not only the number of gut bacterial taxa increases with age, but some taxonomic rearrangement of microbiota composition may also be observed during the ontogeny. The intensive processes of microbiome community formation take place probably shortly after fledging, when fully grown juveniles start to forage independently and their diet gradually becomes similar to the diet of adult birds. However, strong age-related differences in foraging efficiency or foraging habitats are often observed between first-year and older shorebirds, which can maintain some variation in diet long into the postfledging period (e.g. Goss-Custard and Durell 1987, Cresswell 1994). As a result, age-related variation in the gut microbiome diversity and composition may still be detectable during the autumn migration period, as found in this study.

We observed a relatively large number and diversity of putative bacterial pathogens within intestinal tract of all shorebird species migrating through our study sites in Poland. It is well acknowledged that wild birds act as reservoir and vectors of different bacterial and viral diseases (Dhama et al. 2008, Chung et al. 2018). Over the last decades a great effort has been invested to study avian pathogens that cause epidemiologic threat to poultry or humans (e.g. avian influenza virus, West Nile virus, or *Salmonella*) (Brittingham et al. 1988, Martinez-De La Puente et al. 2018). However, an advent of next-generation sequencing era brought an easy access to methodology that allows to detect a broad spectrum of pathogens from different taxonomic groups, providing an unprecedented opportunity to gain a better understanding of interactions between avian hosts and their pathogens (Benskin et al. 2009, Konicek et al. 2016, Rajapaksha et al. 2019, Michel et al. 2021). For example, an application of metabarcoding approaches revealed several potentially pathogenic bacteria in the gut microbiota of two passerine species, Swainson’s thrush *Catharus ustulatus* and gray catbird *Dumetella carolinensis*, migrating along the coast of the Gulf of Mexico, including *Shigella* as the most abundant pathogenic taxon (Lewis et al. 2016). The investigation of the gut microbiota in the Galapagos penguin *Spheniscus mendiculus* revealed the presence of five putative pathogenic taxa, including one taxon (*Clostridium perfringens*) with extremely high prevalence (95% samples) (Rohrer et al. 2023). The analysis of the gut microbiota in three nonpasserine bird species (great bustard, common crane, and common coot *Fulica atra*) during the winter period revealed the presence of 13 potentially pathogenic genera, but all showed rather low (< 4%) relative abundance (Lu et al. 2022). In our study we detected nearly 60 species of putative pathogenic bacteria in faecal samples from shorebirds migrating though Poland and eight of them were found in all study hosts. Moreover, *C. lari, M. iowae*, and *E. cloacae* were present in more than 30% of all sampled individuals (across species). The majority of associations between pathogens and other members of bacterial community were positive, indicating that some nonpathogenic bacterial taxa may promote colonization of digestive tract by pathogenic agents. Taking all this into account, it seems that shorebirds may be considered as relatively important reservoirs of pathogens, which could be primarily associated with their feeding ecology. The preferences for wetland habitats, where the risk of exposure to diverse bacteria is relatively high, especially when pathogens spread through water contaminated by sewage or agricultural practices (Cabral 2010), makes shorebirds specifically prone to act as hosts for a broad spectrum of bacterial pathogens. Between- and within- species transmission of bacterial pathogens may also be promoted by gregariousness during the migratory period, which is typical for most shorebirds, as they usually gather in huge interspecific flocks at favorable stopover sites (van de Kam 2004 et al. 2004, Dhama et al. 2008, Koleček et al. 2021).

The common sandpiper and common snipe were identified as the main reservoirs of *C. lari* in our study, showing significantly higher prevalence than the remaining three shorebird species. In contrast, the dunlin and common sandpiper showed significantly higher prevalence of *E. cloacae* and *V. cholerae* than the common ringed plover, common snipe, and wood sandpiper. Interspecific differences in pathogen prevalence that were observed in our study could be attributed to the divergent foraging techniques or differences in diet between shorebirds, as feeding ecology appears to be a key determinant of bacterial acquisition (Benskin et al. 2009). Our previous research showed that differences in foraging niche could have a major impact on exposure to avian botulism caused by *Clostridium botulinum* in two shorebird species, common snipe and wood sandpiper (Minias et al. 2016). Similarly, the presence of *Campylobacter* bacteria within the intestinal tract was driven by feeding behavior and varied amongst ecological guilds of birds, as insectivores and granivores were only rarely identified as hosts for this pathogen (Waldenström et al. 2002). Despite apparent interspecific variation in the prevalence of some important avian pathogens, an overall species composition of pathogenic gut bacterial fauna was relatively similar between hosts. In consequence, shorebirds are probably exposed to a generally similar array of pathogens and the fine-scale interspecific differences are likely to become more apparent at the level of prevalence, rather than the taxonomic composition of pathogenic bacterial communities.

The differences in prevalence of two bacterial pathogens (*M. iowae* and *V. cholerae*) between stopover sites could be related to local conditions at each stopover site or habitat preferences of migrating individuals. Birds that use locations with high microbial contamination level (e.g. dam reservoirs) could show higher prevalence level of bacterial pathogens than individuals using more natural or less contaminated habitats (e.g. coastal sites). Consistent with this prediction, we observed significantly lower prevalence of *M. iowae* in both common sandpipers and dunlins migrating along the sea coast (< 20%) than through artificial inland reservoir or natural river valley (60%–70%). However, prevalence of *V. cholerae* showed the opposite pattern (higher at the sea coast than inland reservoir), so we acknowledge that there could be many more external or intrinsic factors that shape exposure of shorebirds to pathogens. *Vibrio cholerae* is one of the most important bacterial pathogens in terms of public health, as it can be transmitted to humans, causing acute diarrheal illness (Lipp et al. 2002, Vezzulli et al. 2010). Available information suggest that *V. cholerae* may be occasionally present in wild birds, mostly in species found in aquatic habitats, including shorebirds (Ogg et al. 1989, Hubálek 2004, Ayala and Ogbunugafor 2023). Consistently with these observations, we detected *V. cholerae* in all our study species from different genera, but the prevalence in some species (e.g. 45% in the dunlin) was clearly higher than usually reported for wild birds (Hubálek 2004, Ayala and Ogbunugafor 2023).

In conclusion, our study effectively decomposed taxonomic, environmental (habitat), and intrinsic (age) components of variation in the diversity and composition of gut microbiota among five species of shorebirds. The results indicated that local stopover habitat may play a key role in shaping the gut microbiota of migrating shorebirds, and these local effects may likely dominate over the effects of taxonomic variation. We also provided empirical evidence for the significant role of shorebirds as reservoirs of bacterial pathogens and revealed a complexity of mechanisms that could determine pathogen exposure risk in this group of birds. Thus, our study not only sheds new light on ecological processes that shape avian gut microbiota, but also has implications for our better understanding of host–pathogen interface and the role of birds in long-distance transmission of pathogenic bacteria. At the same time, we acknowledge that any active pathogenicity of gastrointestinal bacteria was not confirmed in our study and, thus, any inferences on the occurrence and role of gut bacterial pathogens in shorebirds should be made with caution.

## Supplementary Material

fiae040_Supplemental_File

## Data Availability

All data are deposited in GenBank Bio-Project: PRJNA1092108.
