# Supplementary material for: Stopover habitat selection drives variation in the gut microbiome composition and pathogen acquisition by migrating shorebirds
Source: FEMS Microbiol Ecol. 2024 Mar 21;100(5):fiae040. doi: 10.1093/femsec/fiae040 (PMC11008731; doi:10.1093/femsec/fiae040)

**Electronic Supplementary Material**

**Stopover habitat selection drives variation in the gut microbiome composition and pathogen acquisition by migrating shorebirds**

**Radosław Włodarczyk^1*^, Joanna Drzewińska-Chańko^1^, Maciej Kamiński^1^, Włodzimierz Meissner^2^, Jan Rapczyński^3^, Katarzyna Janik-Superson^4^, Dawid Krawczyk^5^, Dominik Strapagiel^4^, Agnieszka Ożarowska^2^, Katarzyna Stępniewska^2^, Piotr Minias^1*^**

1. University of Lodz, Faculty of Biology and Environmental Protection, Department of Biodiversity Studies and Bioeducation, Banacha 1/3, 90-237 Łódź, Poland

2. Ornithology Unit, Department of Vertebrate Ecology and Zoology, Faculty of Biology, University of Gdańsk, Wita Stwosza 59, 80-308 Gdańsk, Poland

3. Forestry Student Scientific Association, Ornithological Section, Warsaw University of Life Sciences, Nowoursynowska 166, 02-787 Warszawa, Poland

4. University of Lodz, Faculty of Biology and Environmental Protection, Biobank Lab, Department of Oncobiology and Epigenetics, Pomorska 135, 90-235 Łódź, Poland

5. University of Lodz, Faculty of Biology and Environmental Protection, Department of Invertebrate Zoology & Hydrobiology, Banacha 12/16, 90-237 Łódź, Poland

**Corresponding authors:** University of Lodz, Faculty of Biology and Environmental Protection, Department of Biodiversity Studies and Bioeducation, Banacha 1/3, 90-237 Łódź, Poland

e-mail: radoslaw.wlodarczyk@biol.uni.lodz.pl, e-mail: pminias@op.pl

**TABLE S1.** The number of sequenced faecal samples in five shorebird species migrating through three stopover sites in Poland.

| Species | Stopover site | | | Total |
| --- | --- | --- | --- | --- |
|  | Reservoir | River valley | Sea coast |  |
| Wood sandpiper | 21 | **-** | **-** | 21 |
| Common sandpiper | 13 | 11 | 18 | 42 |
| Common ringed plover | - | 9 | 16 | 25 |
| Dunlin | - | 13 | 18 | 31 |
| Common snipe | 17 | - | - | 17 |
| Total | 51 | 33 | 52 | 136 |

**TABLE S2.** Interspecific variation in two alpha diversity measures, ASV richness (A) and Shannon Index H’ (B) of the gut microbiome across five shorebird species migrating through Poland. The common sandpiper and dunlin were included as reference intercept groups in the analyses of ASV richness and Shannon index, respectively. Sample storage buffer was included as a fixed factor. Significant predictors are marked in bold.

| Predictor | β ± SE | *t* | *P* |
| --- | --- | --- | --- |
| A. ASV richness |  |  |  |
| **Intercept** | **4.040 ± 0.0120** | **212.52** | **<0.001** |
| **Species (CAL ALP)** | **0.361 ± 0.025** | **14.73** | **<0.001** |
| **Species (CHA HIA)** | **0.694 ± 0.024** | **28.70** | **<0.001** |
| **Species (TRI GLA)** | **0.511 ± 0.026** | **19.27** | **<0.001** |
| **Species (GAL GAL)** | **0.410 ± 0.027** | **14.96** | **<0.001** |
| **Buffer (EtOH vs. ATL)** | **1.176 ± 0.016** | **74.56** | **<0.001** |
| B. Shannon index |  |  |  |
| **Intercept** | **3.044 ± 0.206** | **14.77** | **<0.001** |
| Species (ACT HYP) | -0.495 ± 0.256 | -1.93 | 0.055 |
| Species (CHA HIA) | -0.217 ± 0.283 | -0.77 | 0.444 |
| **Species (TRI GLA)** | **-0.778 ± 0.299** | **-2.60** | **0.010** |
| **Species (GAL GAL)** | **-0.642 ± 0.316** | **-2.03** | **0.045** |
| **Buffer (EtOH vs. ATL)** | **0.953 ± 0.217** | **4.39** | **<0.001** |

**TABLE S3.** Habitat and age variation in Shannon index (H’) of the gut microbiome in three shorebird species. Sample storage buffer was included as a fixed factor. Significant predictors are marked in bold.

| Species | Predictor | β ± SE | *t* | *P* |
| --- | --- | --- | --- | --- |
| Common sandpiper | Shannon index |  |  |  |
|  | **Intercept** | **2.354 ± 0.288** | **8.17** | **< 0.001** |
|  | Site (sea coast vs. reservoir) | 0.324 ± 0.363 | 0.89 | 0.377 |
|  | Site (river valley vs. reservoir) | 0.357 ± 0.416 | 0.86 | 0.396 |
|  | Buffer (EtOH vs. ATL) | 0.568 ± 0.534 | 1.06 | 0.294 |
| Dunlin | Shannon index |  |  |  |
|  | **Intercept** | **2.8 ± 0.452** | **6.28** | **< 0.001** |
|  | Site (river valley vs. sea coast) | -0.449 ± 0.590 | -0.76 | 0.453 |
|  | Age (adult vs. juvenile) | 0.518 ± 0.479 | 1.08 | 0.289 |
|  | **Buffer (EtOH vs. ATL)** | **1.279 ± 0.519** | **2.46** | **0.020** |
| Common ringed plover | Shannon index |  |  |  |
|  | **Intercept** | **2.287 ± 0.284** | **8.05** | **<0.001** |
|  | Site (river valley vs. sea coast) | 1.496 ± 0.733 | 2.04 | 0.054 |
|  | Age (adult vs. juvenile) | -0.098 ± 0.409 | -0.24 | 0.812 |
|  | Buffer (EtOH vs. ATL) | 1.086 ± 0.787 | 1.38 | 0.182 |

**TABLE S4.** Putative avian pathogens detected in the gut microbiome of five shorebird species migrating through Poland based on classification provided by Benskin et al. (2009). Prevalence values are reported only for pathogens recorded in >2 % of all samples, further analyses performed only for pathogens with prevalence above 20 %).

| Putative pathogen | Total prevalence | GAL GAL | TRI GLA | ACT HYP | CAL ALP | CHA HIA |
| --- | --- | --- | --- | --- | --- | --- |
| 1. *Campylobacter lari* | 39.0% | 47.1% | 19.1% | 64.3% | 35.5% | 12.0% |
| 2. *Mycoplasma iowae* | 30.9% | 41.2% | 28.6% | 35.7% | 32.3% | 16.0% |
| 3. *Enterobacter cloacae* | 30.9 % | 11.8% | 23.8% | 40.5% | 41.9% | 20.0% |
| 4. *Vibrio cholerae* | 21.3% | 11.8% | 9.5% | 23.8% | 45.2% | 4.0% |
| 5. *Campylobacter jejuni* | 18.4 % | 23.5% | 0.0% | 14.3% | 25.8% | 28.0% |
| 6. *Pseudomonas sp.* | 14.0 % | 29.4% | 14.3% | 7.1% | 12.9% | 16.0% |
| 7. *Enterococcus durans* | 10.3% | 0.0% | 23.8% | 11.9% | 12.9% | 0.0% |
| 8. *Staphylococcus succinus* | 10.3 % | 11.8% | 33.3% | 9.5% | 0.0% | 4.0% |
| 9. *Streptococcus salivarius* | 5.9% | 5.9% | 9.5% | 7.1% | 3.2% | 4.0% |
| 10. *Clostridium botulinum* | 5.1% | 5.9% | 4.8% | 0.0% | 6.5% | 12.0% |
| 11. *Klebsiella oxytoca* | 2.9% | 0.0% | 4.8% | 7.1% | 0.0% | 0.0% |
| 12. *Chlamydia sp.,* 13. *Staphylococcus hominis,* 14. *Streptococcus plutanimalium,* 15. *Streptococcus suis,* 16. *Mycoplasma gallisepticum,* 17. *Campylobacter canadensis,* 18*. Pseudomonas aeruginosa,* 19*. Clostridium chauvoei*  20*. Streptococcus agalactiae,* 21*. Staphylococcus haemolyticus,* 22*. Streptococcus anginosus,* 23. *Staphylococcus pettenkoferi*  24*. Clostridium tyrobutyricum,* 25*. Pseudomonas syringae,* 26. *Klebsiella pneumoniae* | | | | | | |

**TABLE S5.** Putative avian pathogens detected in the gut microbiome of five shorebird species migrating through Poland, as identified based on FAPROTAX classification. Pathogeny of identified bacterial species was based on classification provided by Benskin et al. (2009).

| No | Bacterial species | Number of samples | Confirmed pathogenity | Ecological group | | | |
| --- | --- | --- | --- | --- | --- | --- | --- |
|  |  |  |  | Human pathogens | Intracellular parasites | Animal parasites or symbionts | Predatory or exoparasitic |
| 1 | *Acinetobacter baumannii* | 1 | YES | YES |  | YES |  |
| 2 | *Acinetobacter calcoaceticus* | 5 | YES | YES |  | YES |  |
| 3 | *Aeromonas hydrophila* | 21 | YES | YES |  | YES |  |
| 4 | *Arcobacter butzleri* | 1 | YES | YES |  | YES |  |
| 5 | *Chlamydia sp.* | 1 | YES |  | YES |  |  |
| 6 | *Citrobacter braakii* | 1 | YES |  |  | YES |  |
| 7 | *Citrobacter freundii* | 63 | YES |  |  | YES |  |
| 8 | *Citrobacter rodentium* | 1 | YES |  |  | YES |  |
| 9 | *Citrobacter sp.* | 4 | YES |  |  | YES |  |
| 10 | *Cupriavidus respiraculi* | 1 | NO |  |  |  | YES |
| 11 | *Enterobacter sp.* | 1 | NO |  |  | YES |  |
| 12 | *Enterococcus faecalis* | 8 | YES | YES |  | YES |  |
| 13 | *Entomoplasma freundtii* | 1 | NO |  |  | YES |  |
| 14 | *Escherichia albertii* | 14 | YES |  |  | YES |  |
| 15 | *Escherichia coli* | 77 | YES | YES |  | YES |  |
| 16 | *Estrella lausannensis* | 1 | NO |  | YES |  |  |
| 17 | *Hafnia alvei* | 1 | YES |  |  | YES |  |
| 18 | *Helicobacter pullorum* | 16 | YES | YES |  | YES |  |
| 19 | *Legionella pneumophila* | 4 | NO | YES |  | YES |  |
| 20 | *Legionella sp.* | 1 | NO |  | YES |  |  |
| 21 | *Listonella anguillarum* | 8 | NO |  |  | YES |  |
| 22 | *Mannheimia varigena* | 1 | YES |  |  | YES |  |
| 23 | *Mobiluncus curtisii* | 1 | NO |  |  | YES |  |
| 24 | *Morganella morganii* | 16 | YES | YES |  | YES |  |
| 25 | *Pantoea agglomerans* | 5 | NO | YES |  | YES |  |
| 26 | *Pasteurellaceae bacterium* | 1 | YES |  |  | YES |  |
| 27 | *Providencia alcalifaciens* | 18 | NO | YES |  | YES |  |
| 28 | *Roseomonas terricola* | 14 | NO | YES |  | YES |  |
| 29 | *Serratia marcescens* | 1 | YES | YES |  | YES |  |
| 30 | *Staphylococcus saprophyticus* | 2 | YES | YES |  | YES |  |
| 31 | *Stenotrophomonas maltophilia* | 3 | YES | YES |  | YES |  |
| 32 | *Stenotrophomonas sp.* | 3 | YES | YES |  | YES |  |
| 33 | *Vibrio vulnificus* | 4 | YES | YES |  | YES |  |

**TABLE S6.** Interspecific variation in the prevalence of putative avian pathogens in the gut microbiomes across five shorebird species. The species with the highest prevalence level of each pathogen (see table 4) was included as reference intercept group in all the models. The models were run for putative pathogens with >20% total prevalence. Significant differences are marked in bold.

| Species | β ± SE | *Z* | *P* |
| --- | --- | --- | --- |
| A. *Campylobacter lari* |  |  |  |
| Intercept | 0.544 ± 0.325 | 1.67 | 0.094 |
| GAL GAL | -0.867 ± 0.607 | -1.43 | 0.153 |
| **CAL ALP** | **-1.341 ± 0.521** | **-2.57** | **0.010** |
| **TRI GLA** | **-2.097 ± 0.648** | **-3.23** | **0.001** |
| **CHA HIA** | **-2.694 ± 0.709** | **-3.80** | **<0.001** |
| Buffer (EtOH vs. ATL) | 0.493 ± 0.465 | 1.06 | 0.289 |
| B. *Mycoplasma iowae* |  |  |  |
| Intercept | -1.351 ± 0.616 | -2.19 | 0.028 |
| ACT HYP | 0.550 ± 0.692 | 0.79 | 0.426 |
| CAL ALP | -0.441 ± 0.720 | -0.61 | 0.540 |
| TRI GLA | -0.091 ± 0.789 | -0.11 | 0.909 |
| CHA HIA | -1.284 ± 0.828 | -1.55 | 0.121 |
| **Buffer (EtOH vs. ATL)** | **2.228 ± 0.503** | **4.43** | **<0.001** |
| C. *Enterobacter cloacae* |  |  |  |
| Intercept | 0.608 ± 0.469 | 1.295 | 0.195 |
| ACT HYP | -0.831 ± 0.568 | -1.46 | 0.144 |
| **TRI GLA** | **-1.499 ± 0.705** | **-2.13** | **0.033** |
| **CHA HIA** | **-1.588 ± 0.698** | **-2.27** | **0.023** |
| **GAL GLA** | **-2.030 ± 0.910** | **-2.23** | **0.026** |
| **Buffer (EtOH vs. ATL)** | **-3.394 ± 1.077** | **-3.15** | **0.001** |
| D. *Vibrio cholerae* |  |  |  |
| Intercept | -0.211 ± 0.418 | -0.50 | 0.614 |
| ACT HYP | -0.957 ± 0.535 | -1.79 | 0.074 |
| **GAL GAL** | **-1.823 ± 0.835** | **-2.18** | **0.029** |
| **TRI GLA** | **-2.049 ± 0.833** | **-2.46** | **0.014** |
| **CHA HIA** | **-2.980 ± 1.084** | **-2.75** | **0.006** |
| Buffer (EtOH vs. ATL) | 0.042 ± 0.542 | 0.08 | 0.938 |

**TABLE S7.** Habitat and age variation in the prevalence of *Enterobacter cloacae* in three shorebird species. Significant predictors are marked in bold**.**

| Species | Predictor | β ± SE | *Z* | *P* |
| --- | --- | --- | --- | --- |
| Common sandpiper | Intercept | -1.504 ± 0.782 | -1.92 | 0.054 |
|  | Site (sea coast vs. reservoir) | 1.504 ± 0.928 | 1.62 | 0.105 |
|  | **Site (river valley vs. reservoir)** | **2.064 ± 1.002** | **2.06** | **0.039** |
|  | Buffer (EtOH vs. ATL) | -16.94 ± 1847 | -0.01 | 0.993 |
|  |  |  |  |  |
| Dunlin | Intercept | 0.712 ± 0.989 | 0.72 | 0.473 |
|  | Site (river valley vs. sea coast) | -0.052 ± 1.417 | -0.04 | 0.971 |
|  | Age (adult vs. juv.) | 0.104 ± 1.116 | 0.10 | 0.925 |
|  | Buffer (EtOH vs. ATL) | -20.26 ± 3103 | -0.01 | 0.995 |
| Common ringed plover | Intercept | -1.221 ± 0.726 | -1.68 | 0.093 |
|  | Site (river valley vs. sea coast) | 1.221 ± 1.590 | 0.77 | 0.442 |
|  | Age (adult vs. juv.) | -0.768 ± 1.247 | -0.62 | 0.538 |
|  | Buffer (EtOH vs. ATL) | -1.513 ± 1.823 | -0.83 | 0.407 |

**TABLE S8.** Habitat and age variation in the prevalence of *Mycoplasma iowae* in three shorebird species. Significant predictors are marked in bold**.**

| Species | Predictor | β ± SE | *Z* | *P* |
| --- | --- | --- | --- | --- |
| Common sandpiper | Intercept | -0.470 ± 0.570 | 0.39 | 0.693 |
|  | **Site (sea coast vs. reservoir)** | **-2.079 ± 0.852** | **-2.41** | **0.016** |
|  | Site (river valley vs. reservoir) | -1.030 ± 0.847 | -0.92 | 0.358 |
|  | Buffer (EtOH vs. ATL) | 2.321 ± 1.408 | 1.65 | 0.099 |
|  |  |  |  |  |
| Dunlin | Intercept | -3.047 ± 1.625 | -1.87 | 0.061 |
|  | **Site (river valley vs. sea coast)** | **2.820 ± 1.571** | **1.79** | **0.073** |
|  | Age (adult vs. juv.) | -0.076 ± 1.430 | -0.05 | 0.958 |
|  | Buffer (EtOH vs. ATL) | 1.435 ± 1.195 | 1.20 | 0.230 |
| Common ringed plover | **Intercept** | **-2.933 ± 1.198** | **-2.45** | **0.014** |
|  | Site (river valley vs. sea coast) | -14.63 ± 2797 | -0.01 | 0.996 |
|  | Age (adult vs. juv.) | 0.524 ± 1.241 | 0.42 | 0.673 |
|  | Buffer (EtOH vs. ATL) | 17.05 ± 2797 | -0.01 | 0.995 |

**TABLE S9.** Habitat and age variation in the prevalence of *Vibrio cholerae* in two shorebird species. Significant predictors are marked in bold**.**

| Species | Predictor | β ± SE | *Z* | *P* |
| --- | --- | --- | --- | --- |
| Common sandpiper | **Intercept** | **-2.446 ± 1.055** | **-2.32** | **0.020** |
|  | **Site (sea coast vs. reservoir)** | **2.477 ± 1.143** | **2.17** | **0.030** |
|  | Site (river valley vs. reservoir) | -17.12 ± 3242 | -0.01 | 0.996 |
|  | Buffer (EtOH vs. ATL) | -0.278 ± 1.362 | -0.20 | 0.838 |
|  |  |  |  |  |
| Dunlin | Intercept | -1.707 ± 1.240 | -1.38 | 0.168 |
|  | Site (river valley vs. sea coast) | -19.75 ± 4157 | -0.01 | 0.996 |
|  | **Age (adult vs. juv.)** | **3.569 ± 1.383** | **2.58** | **0.009** |
|  | Buffer (EtOH vs. ATL) | 18.35 ± 4157 | 0.00 | 0.997 |

**TABLE S10.** Habitat and age variation in the prevalence of *Campylobacter lari* in three shorebird species. Significant predictors are marked in bold.

| Species | Predictor | β ± SE | *Z* | *P* |
| --- | --- | --- | --- | --- |
| Common sandpiper | Intercept | 1.135 ± 0.676 | 1.68 | 0.093 |
|  | Site (sea coast vs. reservoir) | -0.966 ± 0.814 | -1.19 | 0.235 |
|  | Site (river valley vs. reservoir) | -0.576 ± 0.922 | -0.62 | 0.532 |
|  | Buffer (EtOH vs. ATL) | 0.505 ± 1.248 | 0.40 | 0.686 |
|  |  |  |  |  |
| Dunlin | Intercept | -1.241 ± 0.992 | -1.25 | 0.211 |
|  | Site (river valley vs. sea coast) | 0.365 ± 1.215 | 0.30 | 0.764 |
|  | Age (adult vs. juv.) | 0.218 ± 1.022 | 0.21 | 0.831 |
|  | Buffer (EtOH vs. ATL) | 0.882 ± 1.034 | 0.85 | 0.394 |
| Common ringed plover | **Intercept** | **-2.591 ± 1.115** | **-2.32** | **0.020** |
|  | Site (river valley vs. sea coast) | -14.975 ± 2.797 | -0.01 | 0.995 |
|  | Age (adult vs. juv.) | 1.308 ± 1.313 | 0.99 | 0.319 |
|  | Buffer (EtOH vs. ATL) | 15.063 ± 2.797 | 0.01 | 0.995 |

**FIGURE S1.** Differences in ASV richness (A) and Shannon index (B) between five shorebird species migrating through Poland. Mean (± SE) residuals from GLM models (Table S2) are presented.


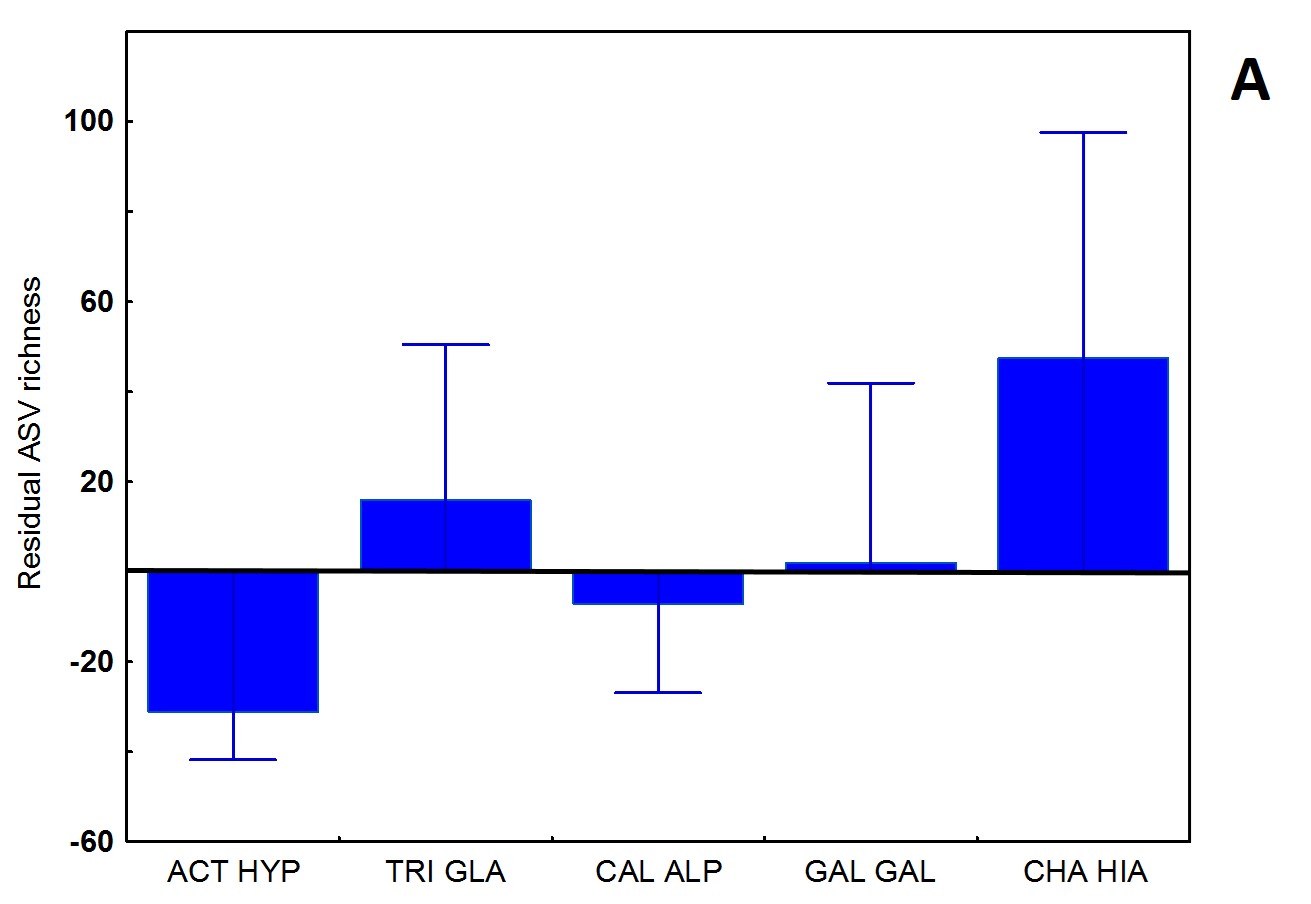


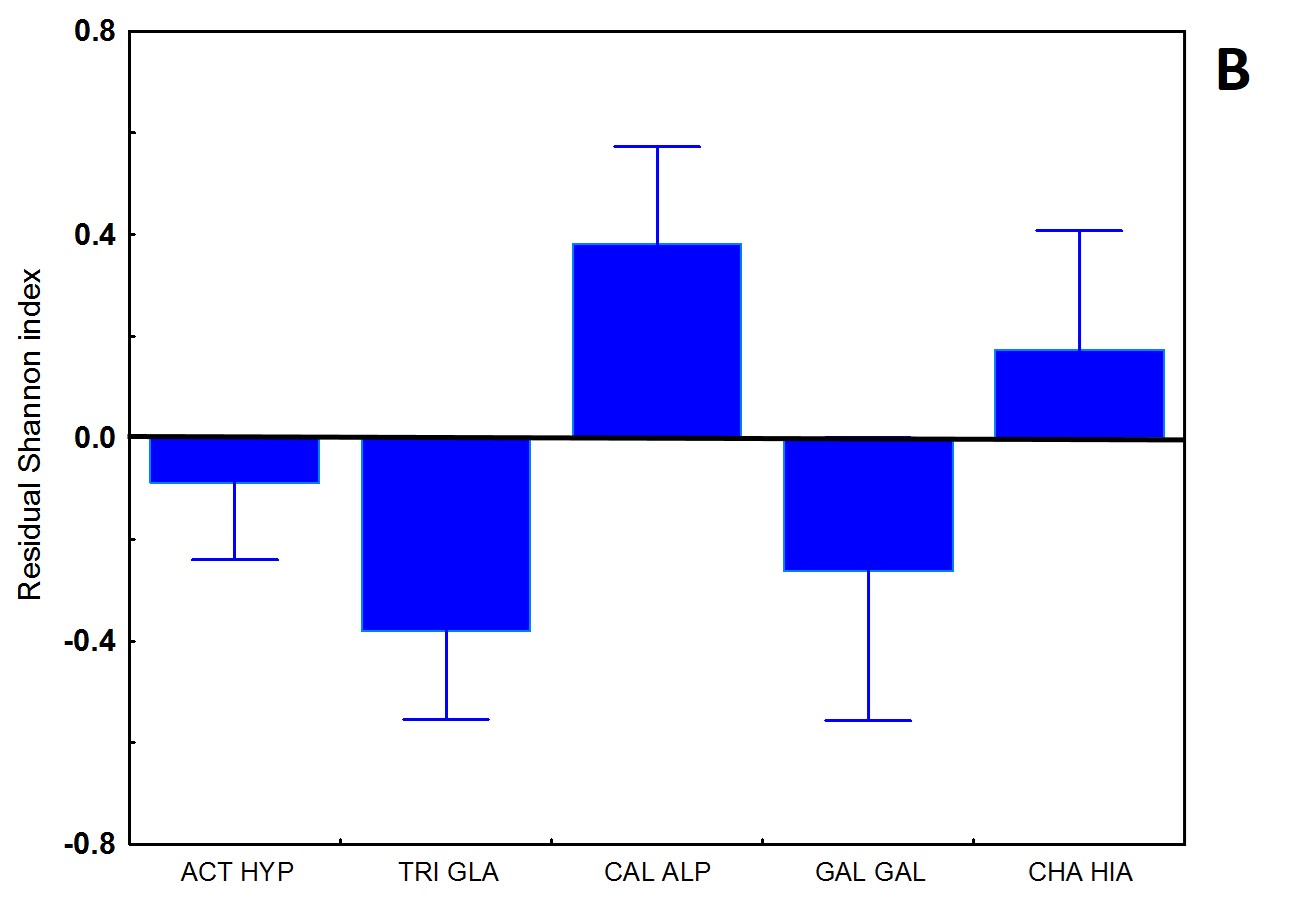


**FIGURE S2**. Non-multidimensional scaling (NMDS) plot based on Jaccard distances showing clustering of shorebird gut microbiota communities by stopover site (shape) and species (colour).

**FIGURE S3**. Associations between interspecific differentiation (pairwise Jaccard distances) in diet and microbiome composition in five shorebird species migrating through Poland. Diet composition was characterized at family (A) and class (B) level.


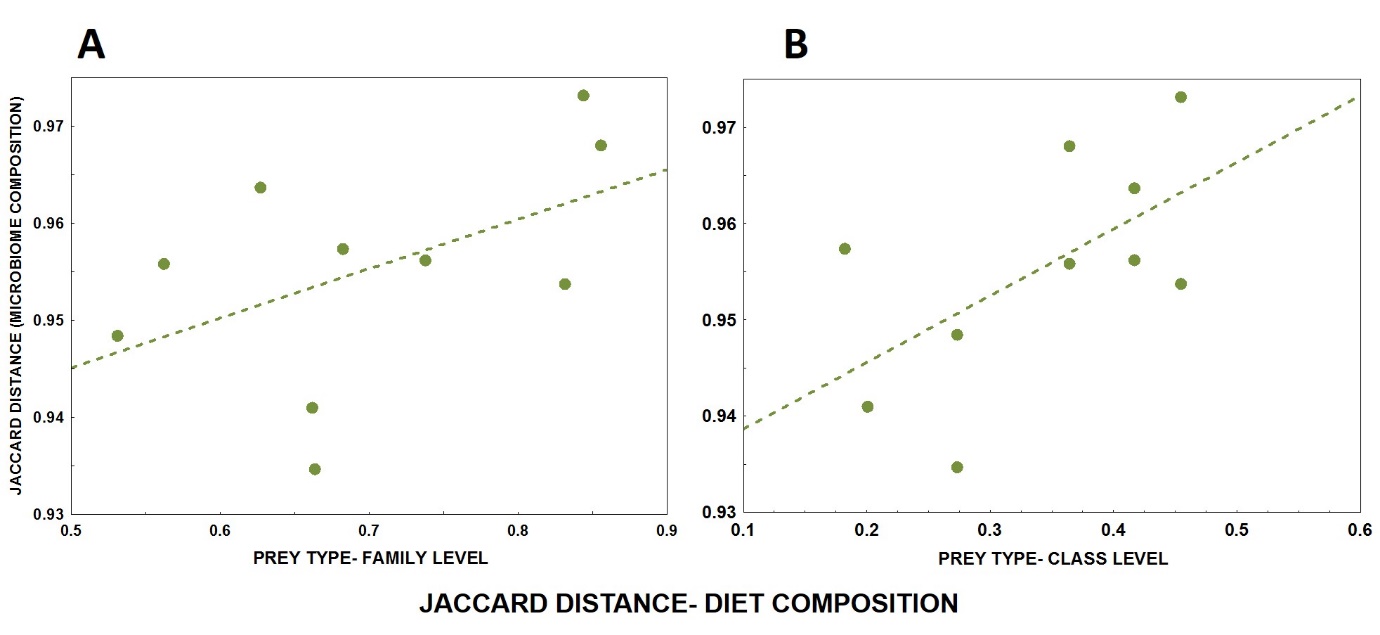


**FIGURE S4**. Co-occurrence network for four major putative pathogenic bacteria detected in the gut microbiome of five shorebird species migrating through Poland (number of inter-connections in brackets).


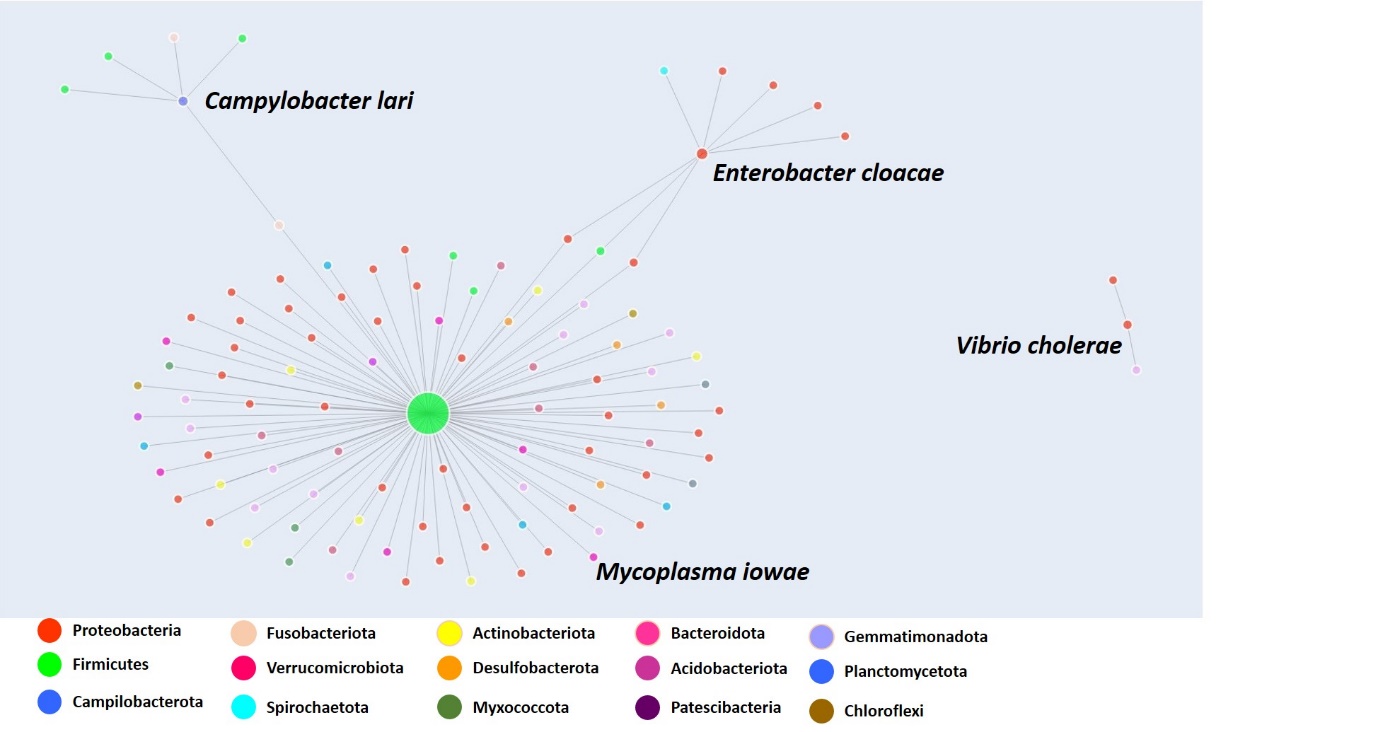

Supplement: fiae040_Supplemental_File [file fiae040_supplemental_file.docx]
